# Herbicide-resistant weeds: from research and knowledge to future needs

**DOI:** 10.1111/eva.12098

**Published:** 2013-09-10

**Authors:** Roberto Busi, Martin M Vila-Aiub, Hugh J Beckie, Todd A Gaines, Danica E Goggin, Shiv S Kaundun, Myrtille Lacoste, Paul Neve, Scott J Nissen, Jason K Norsworthy, Michael Renton, Dale L Shaner, Patrick J Tranel, Terry Wright, Qin Yu, Stephen B Powles

**Affiliations:** 1 School of Plant Biology, University of Western AustraliaPerth, WA, Australia; 2 IFEVA – CONICET – Facultad de Agronomía, Universidad de Buenos AiresBuenos Aires, Argentina; 3 Saskatoon Research Centre, Agriculture and Agri-Food CanadaSaskatoon, SK, Canada; 4 Biology and Logistics, SyngentaBracknell, UK; 5 School of Life Sciences, Warwick HRI, University of WarwickWarwick, UK; 6 Department of Bioagricultural Sciences and Pest Management, Colorado State UniversityFort Collins, CO, USA; 7 Crop, Soil, and Environmental Sciences Department (Weed Science), University of ArkansasFayetteville, AR, USA; 8 Agricultural Research Service, US Department of AgricultureFort Collins, CO, USA; 9 Department of Crop Science, University of IllinoisUrbana, IL, USA; 10 Intellectual Property Portfolio Development, Dow AgroSciencesIndianapolis, IN, USA

**Keywords:** agriculture, global crops, herbicide resistance, population genetics, plant adaptation, weed science

## Abstract

Synthetic herbicides have been used globally to control weeds in major field crops. This has imposed a strong selection for any trait that enables plant populations to survive and reproduce in the presence of the herbicide. Herbicide resistance in weeds must be minimized because it is a major limiting factor to food security in global agriculture. This represents a huge challenge that will require great research efforts to develop control strategies as alternatives to the dominant and almost exclusive practice of weed control by herbicides. Weed scientists, plant ecologists and evolutionary biologists should join forces and work towards an improved and more integrated understanding of resistance across all scales. This approach will likely facilitate the design of innovative solutions to the global herbicide resistance challenge.

Since the late 1940s, synthetic herbicides have been used in agriculture on a global scale to control weeds. As in any perturbed biological environment, herbicide use has resulted in plant evolution and adaptation by the selection of genetic traits conferring phenotypic resistance (i.e., mechanisms protecting plants by a reduction in the herbicide damage) and allowing weedy plants to survive and reproduce in the presence of herbicides. To date, there have been many major contributions to the study of the evolution of weed resistance to herbicides (Powles and Matthews 1991; Powles and Holtum 1994; Jasieniuk et al. 1996; De Prado et al. 1997; Gressel 2000; Powles and Shaner 2001; Tranel and Wright 2002; Délye 2005; Powles and Yu 2010). In February 2013, over 350 delegates from 30 countries convened at the international ‘Global Herbicide Resistance Challenge 2013′ conference (http://www.herbicideresistanceconference.com.au/), convened by Stephen Powles (Australian Herbicide Resistance Initiative, University of Western Australia), to participate in a multidisciplinary forum which focused on the state of knowledge and management of weed resistance to herbicides. Several keynote speakers highlighted the recent progress made on our basic understanding of herbicide-resistant weeds and management of their impact on agro-ecosystems. Here, we summarize the most exciting areas and highlight future challenges of herbicide resistance research toward an integrated and (evolutionary) sustainable weed management.

## Current understanding of herbicide resistance mechanisms

Most herbicides inhibit specific enzymes in plants (target sites of action). Mutations in the target-site genes conferring functional enzymatic insensitivity to herbicides and subsequent target-site resistance (TSR) have been extensively reported (reviewed by Tranel and Wright 2002; Délye 2005; Powles and Yu 2010). Presentations from Qin Yu (University of Western Australia) and Deepak Kaundun (Syngenta, UK) revealed our detailed molecular and biochemical understanding in resistance-endowing mutations in the genes coding for two major herbicide targets, *ALS* and *ACCase*. However, resistance-endowing genes not directly related to specific herbicide targets have also been frequently reported as resistance mechanisms in plants, especially in grasses (Beckie and Tardif 2012). The functional role of these non-target-site resistance (NTSR) genes is to minimize the amount of herbicide that reaches the herbicide site of action so that plants can maintain fitness under herbicide selection. Christophe Délye (INRA, France) emphasized that the present understanding of the genetic basis of NTSR and subsequent molecular identification in plant genomes remains limited, yet, ‘omics’ technologies based on next-generation sequencing are showing the potential to revolutionize the discovery of NTSR genes. In plants, NTSR mechanisms are mediated by stress-response enzymes including complex constitutive and/or induced interactions of cytochrome P450 mono-oxygenase, glutathione *S*-transferase, glycosyltransferase, and/or ATP-binding cassette transporter polygene families (Yuan et al. 2007; Powles and Yu 2010; Delye 2013). NTSR can and does coexist in plants with TSR mechanisms. The likelihood that a NTSR or TSR mechanism is selected in a weed population depends on many factors, such as the herbicide mode of action, its use rate, the site of action, the weed species, the population size, and the environment. Conference participants generally shared the common view that a deeper understanding of the genetic and mechanistic basis of NTSR is a high priority in herbicide resistance research. Thus, a few studies stood out reporting current progress on identification and functional analysis of candidate NTSR genes in *Alopecurus myosuroides* (Gardin, INRA, France) and *Lolium rigidum* (Gaines, University of Western Australia) by high-throughput sequencing. These studies represent the promise of a greater understanding of the role of NTSR genes in complex detoxification pathways associated with herbicide resistance. One of the most immediate and practical outcomes of such work could be the development of PCR-based DNA markers for NTSR screening and detection. Such markers are already commonly used for rapid diagnosis of TSR (Burgos et al. 2012).

Particular emphasis was also given to fundamental research on evolved glyphosate resistance because of the global overreliance on this herbicide (Duke and Powles 2008). A comprehensive review given by Doug Sammons (Monsanto, USA) reported on the great diversity of resistance mechanisms in glyphosate-resistant plants, including reduced glyphosate translocation, rapid leaf necrosis, enhanced vacuolar sequestration, multiple amino acid substitutions in the target-site *EPSPS* gene, and *EPSPS* gene amplification. Multiple mechanisms are sometimes found within a single plant genotype, and novel resistance mechanisms continue to appear. Adam Jalaludin (University of Western Australia) reported on a double point mutation (Thr102Ile and Pro106Ser, known as TIPS) previously engineered in maize and now arising spontaneously in evolved glyphosate-resistant *Eleusine indica*. François Tardif (University of Guelph, Canada) reported on a mechanism endowing glyphosate resistance that involves a light-activated rapid necrosis response (leaf amputation) in *Ambrosia trifida*.

## Current management of herbicide resistance in global agro-ecosystems

Despite research efforts and the knowledge generated from these efforts, weed resistance has continued to evolve. Ian Heap (International Survey of Herbicide Resistant Weeds, USA) reported a steady rate of increase in resistance at the global, regional and field scale, and encompassing diverse ecological conditions, a total of 217 weed species (129 dicots and 88 monocots) have evolved resistance to herbicides (www.weedscience.org). Jason Norsworthy (University of Arkansas, USA) presented the problematic issues of glyphosate-resistant weeds in the USA, where the monochemical management practices fostered by transgenic glyphosate-resistant crops has led to the rapid evolution of major weeds resistant to glyphosate. The most spectacular current example of this is *Amaranthus palmeri*. In the USA, this species has caused the disruption of agricultural systems based on the cultivation of transgenic glyphosate-resistant crops. Farmers are now using less-effective herbicides in combination with cultural, biological, mechanical - and even manual - weed management practices. The future commercialization and adoption of transgenic crops with additional resistance traits for auxinic herbicides such as 2,4-D (currently there are few known cases of dual resistance to 2,4-D and glyphosate) may provide some diversity in herbicide control tactics (Wright et al. 2010). Yet, herbicide use imposes strong selection intensity for weed resistance and any attempt to manage resistance only through herbicide diversity is insufficient (Norsworthy et al. 2012).

The global epidemic of herbicide-resistant weeds needs a radical change in weed management practices to incorporate more diversity and integrated solutions. A present challenge is to develop integrated cropping systems and demonstrate they can be easily implemented, are economically viable, and are more robust than exclusive herbicidal weed management. Michael Walsh (University of Western Australia) reviewed the use of nonherbicidal techniques targeting weed seeds during crop harvest. In Australia, new integrated tools have continued to be developed to target herbicide-resistant weeds and adapted to the system (i.e., the Harrington Seed Destructor; Walsh et al. 2012). Thus, long-term research and extension efforts toward the integration of these methods have contributed to sustainable herbicide resistance management and profitable farming.

## Research challenges to advance knowledge and management of resistance

Research is essential to develop integrated control strategies as alternatives to the dominant and almost exclusive practice of weed control by herbicides in global field crops. Paul Neve (University of Warwick, UK) highlighted the need to consider weed resistance research within an evolutionary ecological context. The application of evolutionary principles to agricultural settings is not new, but it is of crucial importance to understand and manage the effect of herbicide selection intensity with a system perspective (Thrall et al. 2011). Our deep and sophisticated understanding of the molecular, biochemical, and physiological bases of herbicide resistance at the genetic and cellular level (in essence a description of the consequences of selection) has often failed to illuminate the interpretation of the evolutionary and ecological aspects of herbicide resistance evolution (Neve 2007). Martin Vila-Aiub (University of Buenos Aires, Argentina) re-emphasized how a greater understanding of the causes, dynamics, and processes of resistance evolution could be gained by studies that assess the adaptive value of selected herbicide resistance alleles. The ability of resistant weeds to persist, reproduce, and invade new selective environments depends on the fitness level of the resistant gene (in both the absence and presence of herbicide selection; Maynard Smith 1998). A greater focus to assess the effects of the environment (temperature, abiotic stresses, etc.) on the fitness of resistant plants under current and future cropping conditions could identify conditions that broadly decrease heritability and frequencies of resistance alleles over time (Vila-Aiub et al. 2013). Yet, no studies have systematically addressed the effects of climate change on herbicide resistance evolution. In this regard, Michael Renton (University of Western Australia) showed that individual-based and spatially explicit computational modeling approaches should be more widely adopted to explain such complexities (Renton 2013). These tools could be useful to allow scientists to make more robust predictions on how genetics, plant and seed biology, spatial structure in populations, environmental conditions, and different management options all interact to affect the evolutionary dynamics of weed resistance.

Dale Shaner's (USDA, USA) perspective summarized the obstacles to weed resistance management by setting future research challenges to integrate our understanding at different scales. In the last two decades, modern agricultural systems have been characterized by a lack of diversity in management practices and herbicide over-reliance, in concert with limited herbicide discoveries. Analogous to the rapid evolution of multidrug resistance phenotypes among human pathogens (Alonso et al. 2001), this scenario has led to a rapid global increase in multiple-resistant weed populations with enhanced capacity for herbicide metabolism. Because of the significance of enhanced herbicide metabolism as a resistance mechanism, it was suggested that routes of herbicide metabolism could guide a new classification and ranking of the risk of herbicide resistance evolution. This would serve, in addition to the current classification by herbicide mode of action, as a tool to devise effective herbicide rotations (i.e., rotations based on both site of action and metabolism route). Inevitably, to fully implement this, more research is needed to better understand the molecular players in herbicide metabolism.

## Conclusions

The study of herbicide resistance has shown that plants employ and can evolve a fascinating biological arsenal for their defense. The unraveling of the complexities in NTSR mechanisms, particularly metabolic-based resistance, is a challenge that has the potential to cause a paradigm shift in our understanding and management of weed resistance. For example, this should improve the efficiency of resistance diagnosis, the knowledge of complex detoxification pathways associated with NTSR and the capacity to design successful treatment strategies for multiple targets. Fundamental research on the mechanistic and genetic basis of resistance must contribute to the search for general processes linking the genetic basis to the evolutionary path to herbicide-resistant plants at different scales: genotypic, population, and ecosystem level. Future research should integrate questions about standing genetic variation versus *de novo* resistance mutations, fitness benefits, and costs under herbicide selection and links between metabolic resistance and general detoxification pathways involved in stress-response dynamics. Advances in technology will provide new tools and climate change could have significant impacts on weed management in global field crops, yet an improved and more integrated understanding of resistance across all scales will be the key to facing the global herbicide resistance challenge.
